# Biologically plausible gated recurrent neural networks for working memory and learning-to-learn

**DOI:** 10.1371/journal.pone.0316453

**Published:** 2024-12-31

**Authors:** Alexandra R. van den Berg, Pieter R. Roelfsema, Sander M. Bohte

**Affiliations:** 1 Machine Learning Group, Centrum Wiskunde & Informatica, Amsterdam, The Netherlands; 2 Department of Vision & Cognition, Netherlands Institute for Neuroscience, Amsterdam, The Netherlands; 3 Department of Integrative Neurophysiology, Center for Neurogenomics and Cognitive Research, Vrije Universiteit Amsterdam, Amsterdam, The Netherlands; 4 Department of Neurosurgery, Academic Medical Center, Amsterdam, The Netherlands; 5 Laboratory of Visual Brain Therapy, Institut National de la Santé et de la Recherche Médicale, Institut de la Vision, Sorbonne Université, Centre National de la Recherche Scientifique, Paris, France; 6 Swammerdam Institute of Life Sciences, University of Amsterdam, Amsterdam, The Netherlands; Institute of Psychology Chinese Academy of Sciences, CHINA

## Abstract

The acquisition of knowledge and skills does not occur in isolation but learning experiences amalgamate within and across domains. The process through which learning can accelerate over time is referred to as learning-to-learn or meta-learning. While meta-learning can be implemented in recurrent neural networks, these networks tend to be trained with architectures that are not easily interpretable or mappable to the brain and with learning rules that are biologically implausible. Specifically, these rules have often employed backpropagation-through-time, which relies on information that is unavailable at synapses that are undergoing plasticity in the brain. Previous studies that exclusively used local information for their weight updates had a limited capacity to integrate information over long timespans and could not easily learn-to-learn. Here, we propose a novel gated memory network named RECOLLECT, which can flexibly retain or forget information by means of a single memory gate and is trained with a biologically plausible trial-and-error-learning that requires only local information. We demonstrate that RECOLLECT successfully learns to represent task-relevant information over increasingly long memory delays in a pro-/anti-saccade task, and that it learns to flush its memory at the end of a trial. Moreover, we show that RECOLLECT can learn-to-learn an effective policy on a reversal bandit task. Finally, we show that the solutions acquired by RECOLLECT resemble how animals learn similar tasks.

## Introduction

A hallmark of human intelligence is the capacity to accumulate knowledge across learning experiences. This capacity not only accelerates learning within one domain, but can also facilitate learning in related domains, a phenomenon referred to as learning-to-learn [1,2]. Standard neural network models lack this ability and quickly and catastrophically forget previously acquired knowledge when they are trained on a new task [3,4]. This is particularly problematic in the case of reversal learning [5], where stimuli are initially associated with a certain reward probability, e.g. stimulus A with a 75% chance of reward and stimulus B with a 25% chance of reward. When the stimulus-reward associations are reversed, i.e. stimulus A is now rewarded with 25% probability and stimulus B with 75% probability, the network has to fully change its weight structure to adjust to the new reward probabilities. To overcome this limitation, researchers have developed meta-learning models that acquire a set of weights over the course of several similar tasks that facilitate generalisation to novel tasks if they bear similarities to previously learned tasks.

Meta-learning can be achieved using various approaches [6–8]. An approach that is plausible from a biological perspective uses recurrent neural networks that are trained with reinforcement learning [9,10]. These networks are trained on a distribution of tasks and learn to rely on information about previous stimuli, actions and rewards to represent the appropriate task context. Subsequently, they can carry out new tasks even if the weights of the network are fixed, provided meta-learning was successful. In this framework, the network learns to accumulate information about the new task in its working memory by observing the reward structure. A previous study by Wang et al. [9] suggested that learning-to-learn could rely on interactions between the prefrontal cortex, the basal ganglia and the thalamus for the build-up of working memory representations that support learning-to-learn. Task switching can happen within one or a few trials by adapting the activity pattern in working memory as opposed to going through the elaborate process of retraining the network connectivity.

Even though the behaviour of these meta-learning models is similar to that of animals, the architectures and learning rules have limited biological plausibility for at least two reasons. Firstly, some of the previous studies on meta-learning relied on complex units, such as the long short-term memory (LSTM) unit [11]. The LSTM unit has three multiplicative gates that control its activity, which is unnecessary for some tasks [12,13], can be difficult to interpret and may not be found in biological neurons. Simplifications of LSTM units have been proposed, such as the gated recurrent unit (GRU), which has two gates [14], and more recently, the light-gated recurrent unit (Light-GRU) with a single gate [12]. Models with these simpler units have yielded good or even superior performance on some tasks compared to architectures with LSTM units [12,14].

Secondly, previous models were trained with non-biological learning rules, such as backpropagation-through-time (BPTT). Updates in BPTT rely on information that is not available locally at synapses (i.e. it is non-local in time [15]). An example of an algorithm that is biologically plausible is AuGMEnT, because synapses trained with this learning rule have access to the necessary information [16]. AUGMEnT includes units with persistent activity for working memory and uses synaptic traces, local signals that are stored within synapses to influence plasticity (information about AuGMEnT can be found in Methods). These traces determine which synapses should be strengthened and which ones should be weakened and help to solve a spatial and a temporal credit assignment problem. The spatial credit assignment problem is related to identifying the synapses in the network that are responsible for the outcome of an action. AuGMEnT solves the spatial credit assignment problem with an attentional feedback signal originating from the selected action that highlights the synapses that are responsible for it and are therefore eligible for plasticity. The temporal credit assignment problem is to identify actions that are associated with rewards that only come after a delay and that may be contingent on later actions. AuGMEnT solves the temporal credit-assignment problem by computing a reward-prediction error and by including memory units, which can maintain information about previous sensory inputs. However, AuGMEnT lacks mechanisms for forgetting and the memory therefore needs to be reset after each trial. The inability to integrate information across trials hinders its ability to learn-to-learn. A related biologically inspired learning rule is e-prop [17], which also approximates BPTT by using synaptic traces.

In this study, we propose RECOLLECT, a learning rule based on Light-GRUs that modifies synapses based exclusively on information that is both local in space and time, making it biologically plausible. RECOLLECT adapts the synaptic tags and traces from AuGMEnT [16] to implement a learning rule that closely approximates BPTT but that can also forget information that is no longer relevant and solves the spatial credit-assignment signal for deeper networks. We show that RECOLLECT can flexibly use its working memory to perform a pro-/anti-saccade task and that it learns-to-learn on a reversal bandit task. Finally, we illustrate similarities between the training of networks with RECOLLECT and how animals acquire these tasks.

## Results

### Architecture

#### Feedforward processing

Our aim is to develop a biologically plausible architecture that can learn to memorise and forget. Specifically, we strived for a brain-like architecture and a learning rule in which all the information necessary for a weight change is available locally, at the synapse.

**Fig 1:**
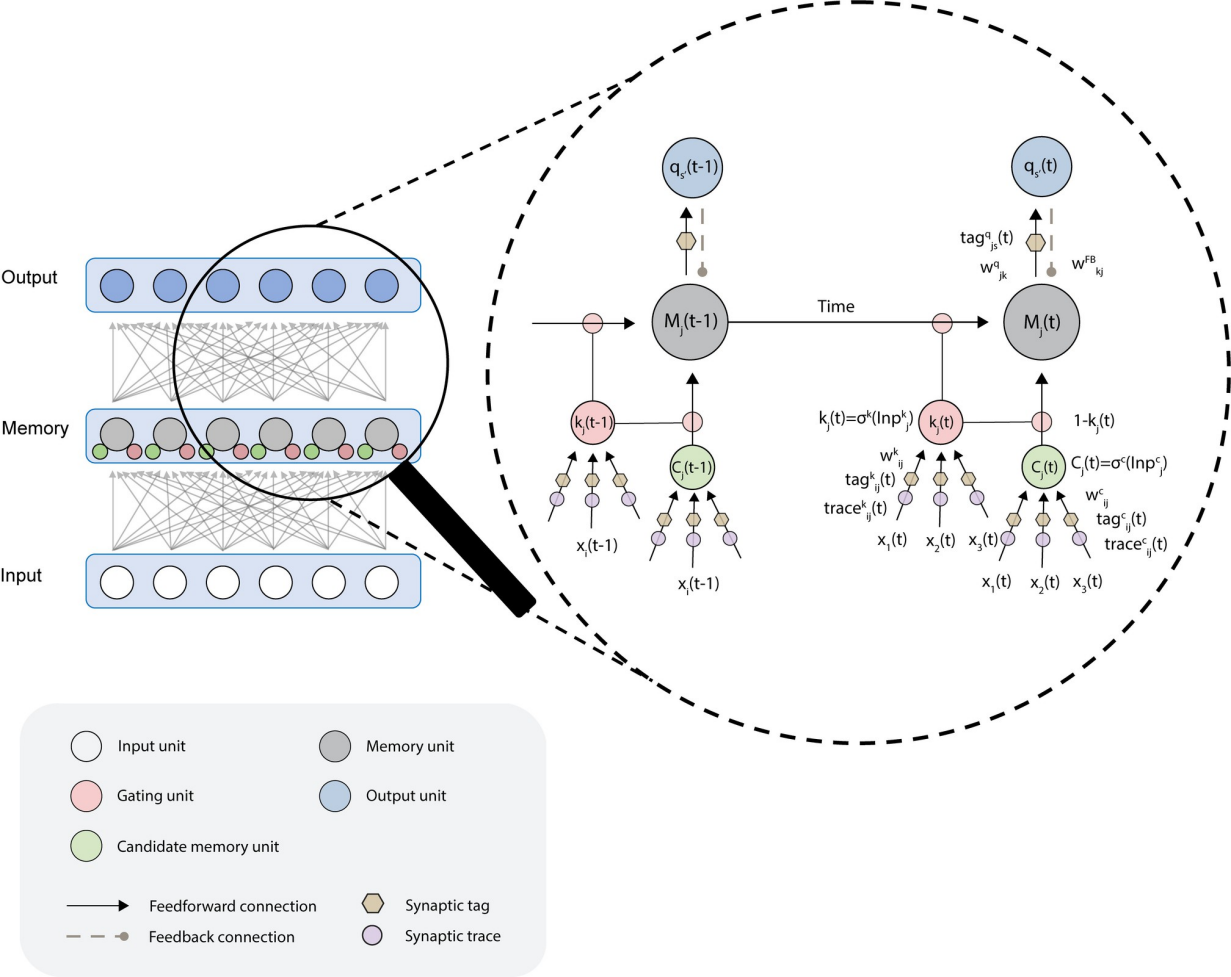
RECOLLECT architecture. Gating units (red circles, k_j_) balance between memory and updating by novel information from candidate memory cells (green circles, C_j_). Memory units (gray circles, M_j_) activate output units that estimate the Q-values of actions (blue circles). Synaptic tags (yellow hexagons) and traces (purple circles) store information that is necessary for the synaptic updates. Traces measure the influence of a connection on the activity of the memory unit and tags the influence of a connection on the selected Q-value unit (see Fig 2A for a more detailed explanation). Dashed grey lines, feedback connections from output units to memory units ($W_{kj}^{FB}$).

The novel model is called “REinforCement learning of wOrking memory with bioLogically pLausible rECurrent uniTs”—RECOLLECT (Fig 1). RECOLLECT draws inspiration from two models: the light-gated recurrent unit (Light-GRU [12]) and AuGMEnT ([16]; see ‘AuGMEnT model’ in Methods). The network’s goal is to learn action-values (known as *Q*-values [18]), which correspond to the amount of reward that is predicted for a particular action when executed in a particular state of the world. If the outcome deviates from the reward-prediction, a neuromodulatory signal that encodes the global reward-prediction error (RPE) gates synaptic plasticity to change the *Q*-value, in accordance with experimental findings [19–22]. RECOLLECT uses a variant of Light-GRU units to learn tasks that require memorisation and forgetting, so that the network can integrate feedback from the environment across trials and determine if it is time to switch to another stimulus-response mapping.

The Light-GRU [12] is a recurrent network that combines incoming sensory information with a memory of the state of the environment of the previous timestep. The maintenance of information in working memory is regulated by a learnable ‘gate’ that determines the influence of the memory and new sensory inputs. This ability enables the network to maintain memories when needed, but also to erase them and focus on new input when memories lose relevance or when the environment changes. Light-GRU units might correspond to a circuit with several neurons in the brain, for example, the neurons of the so-called direct and indirect pathways, which form a loop from cortex to basal ganglia, thalamus and then back to cortex (see Discussion).

RECOLLECT consists of an input layer, a memory layer with GRUs and an output layer. As in Light-GRU [12], the memory layer contains three types of units: candidate memory cells (*C*_*j*_), gating units (*k*_*j*_) and memory cells (*M*_*j*_), which might be part of the same cortical column or part of a loop involving the cortex, basal ganglia and thalamus. Incoming sensory information (*x*_*i*_(*t*)) is processed by the candidate memory cells and available to update the activity of the memory cell:

$$C_{j}(t)=\sigma (\sum_{i}W_{ij}^{C}x_{i}(t)+b_{j}^{C}).$$ (1)

Here, *C*_*j*_ represents the activity of the candidate memory units, $W_{ij}^{C}$ denotes the synaptic weights between sensory unit *i* and candidate memory unit *j*, $b_{j}^{C}$ the bias and *σ*(·) is the sigmoidal activation function used to constrain the output between 0 and 1 (see Eq M1 in the Methods):

The gating units *k*_*j*_ determine the degree to which the memories are maintained or overwritten by new sensory input. The activity of the gating units *k*_*j*_ depends on the input through weights $W_{ij}^{k}$:

$$k_{j}(t)=\sigma (\sum_{i}W_{ij}^{k}x_{i}(t)+b_{j}^{k}).$$ (2)

**Fig 2:**
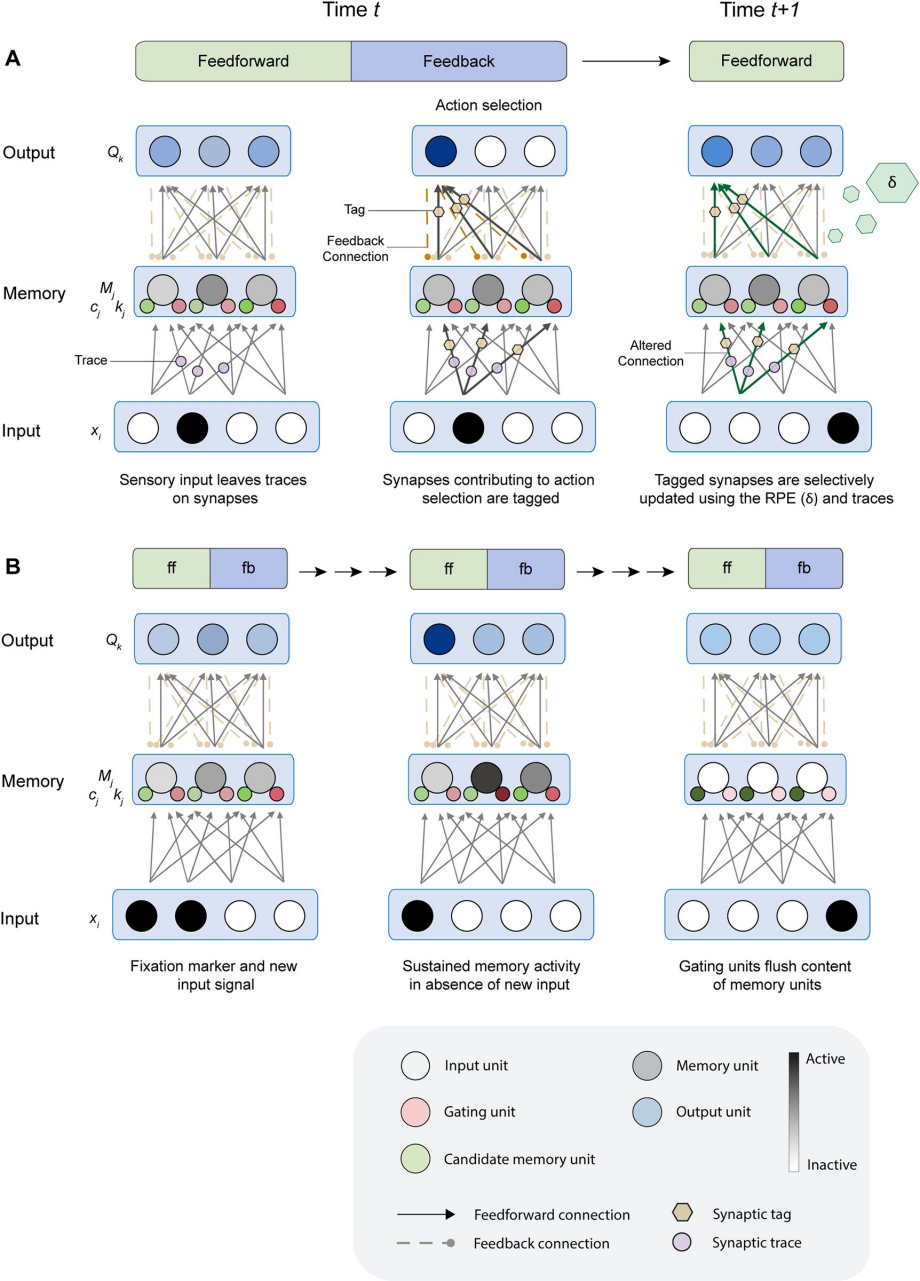
The process of learning and remembering in RECOLLECT. A) Formation of synaptic tags and traces. The activation of input units during feedforward processing creates synaptic traces (purple circles) on the connections to gating and candidate memory units. Upon action selection, relevant synapses contributing to the selected actions are tagged (yellow hexagons) by feedback connections. The RPE is released in the form of a global neuromodulator (green hexagons) when the expected reward based on the Q-value of the selected action is different from the actual reward that is received. The tagged synapses are either potentiated or depressed depending on the sign of the RPE. If the reward is higher or lower than expected, the tagged connections are potentiated or depressed, respectively. B) RECOLLECT flexibly remembers or forgets across multiple time steps, each with a feedforward and feedback phase (as shown in A). Memory units increase their activity when new sensory information is acquired. This activity can be sustained over a memory delay if the gating units (small red circles) are active (dark red colour). When a relevant sensory stimulus is shown at the beginning of the trial it can therefore be memorized. Signals that demarcate the end of a trial can decrease the activity of gating units, causing forgetting (light red colour). Dashed lines indicate feedback connections from output units to memory units.

The gating units determine the updating of the activity of memory units *M*_*j*_ as follows:

$$M_{j}(t)=k_{j}(t){ʘM}_{j}(t-1)+{\left(1-k\right)}_{j}(t))ʘC_{j}(t),$$ (3)

where ʘ refers to element-wise multiplication. If gating units are active, the candidate memory cells do not have much influence on the memory unit and the previous memory *M*_*j*_(*t*−1) is retained. In contrast, if the gating units are only weakly active, the memory units make a large step in the direction of the activity level *C*_*j*_ of the candidate memory cells. We therefore refer to this gate as a memory gate. The process by which RECOLLECT uses memory gates to balance memorisation and forgetting is depicted in Fig 2B.

One important difference between the Light-GRU units in RECOLLECT and those originally formulated by [12] is the exclusion of recurrent weights that allow previous memory states to affect the updating of the gate and candidate memory (Eqs 1 and 2). As we discuss in the next section (‘learning rule’), this allowed us to derive an exact alternative for BPTT, rather than an approximation thereof. Another advantage was the additional simplicity provided to the model. Other differences include a different activation function (sigmoid, rather than rectified linear units) and the exclusion of batch normalisation.

The activity of the memory units is propagated to the output units:

$$q_{k}(t)=\sigma (\sum_{j}W_{jk}^{q}M_{j}(t)+b_{k}^{q}).$$ (4)

The output units estimate the Q-value *q*_*k*_, the expected (discounted) reward of each action *k* that can be taken by the network. Once these values have been computed, an epsilon-greedy strategy selects the winning action *s*, where the action with the highest Q-value is chosen with probability 1-ε, and a random action is selected with probability ε.

Finally, there are feedback connections extending ($W_{sj}^{FB}$) from the output units back to the memory units. As we will discuss in the next section, these feedback connections influence plasticity of connections from input units to gating- and candidate memory units.

#### Learning rule

*Reinforcement learning*. RECOLLECT defines a learning rule for the Light-GRUs that is based on synaptic tags and traces and relies exclusively on information local to the synapse. This rule is equivalent to BPTT when the model does not use recurrent connections (as in the model described in the previous section). In this section, we explain the equations that determine learning in RECOLLECT.

As is common in models of reinforcement learning that use Q-learning, RECOLLECT selects an action *s*, and it may or may not receive a reward. If this reward differs from the expected reward based on the Q-value of the chosen action, this discrepancy gives rise to a reward prediction error (RPE) *δ*:

$$\delta (t)=r(t)+\gamma q_{s}(t)-q_{a}(t-1).$$ (5)

The SARSA temporal difference learning rule compares the predicted outcome of the previous action *q*_*a*_*(t-1)* to the sum of the observed reward *r(t)* and the discounted Q-value of the winning unit *q*_*s*_(*t*). The reward discount factor *γ*, which ranges between 0 and 1, controls the discounting of future rewards, which are considered less valuable than immediate rewards. A negative RPE indicates that the outcome was worse than anticipated, whereas a positive RPE signals that a higher reward was received than was estimated at the previous time step. The RPE is presented to the network in the form of a global neuromodulator, hence it is a signal that is accessible for all synapses in the network.

*Tags and traces*. When synapses are exposed to the neuromodulator that reflects the RPE, plasticity can occur. As in AuGMEnT [16], plasticity is regulated using tags and traces. It is important to distinguish between the role of these components. Tags are formed on all synapses that contributed towards action selection and they register how much a synapse contributed to the selected action [18,23]. Tags also form on the synapses from the input layer to the memory layer, based on feedback connections from the selected action to the memory layer. After their formation, the tags interact with the global neuromodulator that provides information about the RPE. Consequently, only those synapses that were tagged will become plastic.

Because the plasticity rule for feedback connections from the output units to the memory units is the same as that of feedforward connections from the memory units to the output units, these connections become proportional in strength as learning progresses.

Unlike tags, the synaptic traces are only maintained on connections from input units to the candidate memory units and gating units. The synaptic traces measure the influence of a synapse on the activity level of a memory unit, taking the history of memory activity into account. Specifically, if input unit *i* contributed to the activation of a memory unit *j*, then the *trace*_*ij*_ keeps track of how much of this input is still visible in the memory activity, even if this input occurred in the past.

The tags and traces ensure that all the information that is required for network updates is locally available (see Fig 2A for a schematic illustration of the learning rule). The following equations define the updates for the tags, traces and weights for each of the units in RECOLLECT.

For the output units, the tags are formed in the presence of both presynaptic activity (*M*_*j*_) and postsynaptic activity after an action *s* is selected. The ${Tag}_{jk}^{q}$ only increases if the output unit *k* is selected, i.e. if *k* = *s*, in which case the presynaptic activity *M*_*j*_ of memory unit *j* is added to the tag:

$${Tag}_{js}^{q}(t)=\lambda \gamma {Tag}_{js}^{q}(t-1)+M_{j}(t)$$ (6)

$${Tag}_{jk}^{q}(t)=\lambda \gamma {Tag}_{jk}^{q}(t-1);k\neq s$$ (7)

Once a tag is formed, it decays according to two hyper-parameters: the tag decay rate (*λ*) and the aforementioned reward discount factor (*γ*; this parameter is identical to the one used for calculating the RPE in Eq 5). As a result, synapses contributing to previous actions can still be affected by network updates in subsequent timesteps, but to a smaller extent as time progresses. This aspect of the learning scheme corresponds to the temporal difference TD(*λ*) algorithm [24].

*Weight update for output units*. The weight update $\Delta W_{jk}^{q}$ depends on the tag, the RPE *δ* and the learning rate (*β*):

$$\Delta W_{jk}^{q}=\beta \delta (t){Tag}_{jk}^{q}(t).$$ (8)

*Weight update for candidate memory units*. The update of the synapses $W_{ij}^{c}$ from the sensory inputs to the candidate memory cells, providing new input to the memory units, also depend on the degree to which these input cells contributed to Q-value *q*_*s*_ of the selected action *s*. Their influence is indirect, through the memory unit *j*. Plasticity therefore depends on (i) how much memory unit *j* contributed to the Q-value of the selected action and (ii) the contribution of this synapse on the memory unit *j*’s activity level on the current and previous time steps.

The first of these components is reflected by the feedback connection from the selected action, since feedforward and feedback connections between memory and output units are proportional in strength.

The second component is provided by a synaptic trace. Namely, RECOLLECT (as in AugMEnT [16]) uses a ‘trace’ to keep track of the synapse’s influence on the activity level of memory unit *j*. We will first describe the properties of the trace before explaining how it combines with the feedback signal from the selected action to create the tag, which together with the RPE determines the synaptic changes.

The trace measures the influence of an input unit on the activity of a candidate memory cell. It is initialized at a value of 0:

$${Trace}_{ij}^{C}(0)=0$$ (9)

The influence of the synapse $W_{ij}^{C}$ on the activity of memory unit *j* depends on the slope of the activation function $\sigma^{\prime }\left({Inp}_{j}^{C}(t)\right)\;({Inp}_{j}^{C}(t)$ is defined in Eq 1) of the *C*_*j*_ unit at time *t*, the activity of the input unit *x*_*i*_, and on the activity of the memory gate *k*_*j*_, which together define the second term in this equation:

$${Trace}_{ij}^{C}(t)={k_{j}(t)Trace}_{ij}^{C}(t-1)+{[1-k_{j}(t)]x}_{i}(t)\sigma^{\prime }({Inp}_{j}^{C}(t)).$$ (10)

The first term represents a trace of the influence of the synapse on the activity of memory unit *j* on previous time steps ${Trace}_{ij}^{C}(t-1)$. The trace of previous influences quickly declines if *k*_*j*_(*t*) is small, i.e. if the memory gate is open for new sensory input. If the gate activity is close to 1, the memory is maintained and the same holds for the trace. Note that the trace can be computed locally at the synapse and is used to update the tag at the same synapse.

We can now determine the influence of the trace on the tag, which measures the influence of synapse on the current Q-value estimate *q*_*s*_, as follows:

$${Tag}_{ij}^{C}(t)=\lambda \gamma {Tag}_{ij}^{C}(t-1)+{Trace}_{ij}^{C}(t)W_{sj}^{FB}.$$ (11)

Note that the second term includes $W_{sj}^{FB}$, which equals the feedback that arrives at the memory unit *j* through the feedback connection from the winning output unit *s*. This attentional feedback signal is proportional to the contribution of unit *j* to the Q-value of the selected action. The first term implements TD(*λ*) in case *λ* is larger than 0, just as was described above for the weights between the memory units and the output layer.

The ${Tag}_{ij}^{C}$ interacts with globally released neuromodulator that signals the RPE *δ* to determine the weight update, as was also described above:

$$\Delta W_{ij}^{C}=\beta \delta (t){Tag}_{ij}^{C}$$ (12)

Hence, all signals necessary for this weight update are available locally at the synapse.

#### Weight update for gating units

We will now consider the plasticity of the connections of the gating units, which are updated equivalently, using tags and traces. The trace is initialized at time 0:

$${Trace}_{ij}^{k}(0)=0.$$ (13)

The contribution of the synapse $W_{ij}^{k}$ to the activity of the memory unit *j* depends on the slope of the activation function $\sigma^{\prime }\left({Inp}_{j}^{k}(t)\right)$ (with ${Inp}_{j}^{k}(t)$ as defined in Eq 2), the activity of the input unit *x*_*i*_, as well as the difference between the activity of the memory unit at the previous time step *M*_*j*_(*t*-1) and the new input to the memory unit *C*_*j*_, because the activity of the memory gate is irrelevant if the activity of the candidate memory unit is equal to that of the memory unit on the previous time step (as reflected in the second term in Eq 14 below). The first term in the equation below represents the influence of the synapse on the activity of the memory unit on previous time steps, ${Trace}_{ij}^{k}(t-1).$


$${Trace}_{ij}^{k}(t)={k_{j}(t)Trace}_{ij}^{k}(t-1)+{[M_{j}(t-1)-C_{j}(t)]x}_{i}(t)\sigma^{\prime }({Inp}_{j}^{k}(t)).$$ (14)

The equations for the tag and the weight update are equivalent to those of the connections to the candidate memory units (Eqs 11 and 12):

$${Tag}_{ij}^{k}(t)=\lambda \gamma {Tag}_{ij}^{k}(t-1)+{Trace}_{ij}^{k}(t)W_{sj}^{FB}.$$ (15)

$$\Delta W_{ij}^{k}=\beta \delta (t){Tag}_{ij}^{k}.$$ (16)

### Biological plausibility

RECOLLECT uses only local information in its learning rule and has various other properties that were inspired by neurobiology. For instance, the output units in RECOLLECT encode for the Q-value of actions. Neurons coding for action values have been observed in several regions, including the midbrain [22], basal ganglia [25,26] and frontal cortex [27–29].

Moreover, to shape plasticity RECOLLECT makes use of a global neuromodulatory signal that conveys the RPE. Such prediction errors are believed to be generated by midbrain dopamine neurons and support decision-making and learning [30]. Another relevant signal is the sensory prediction error [31]. Eq 14 includes a comparison between the memory unit activity and the current candidate memory unit [*M*_*j*_(*t*−1)−*C*_*j*_(*t*)], representing such a sensory prediction error. Other biological features include the tags (also known as eligibility traces), which are used to demarcate synapses that contribute to the winning unit [32,33]. The tag/tracing mechanism is based on neurophysiological findings, such as the influence of neuromodulators and feedback connections on plasticity (reviewed by [34]). The learning rule represents a form of Hebbian plasticity [35] that depends on both presynaptic and postsynaptic activity, in combination with the RPE.

In conclusion, RECOLLECT is a biologically inspired model that is equipped with a gated memory that allows for selective forgetting and integration of information over longer timespans. In the Methods section we demonstrate that RECOLLECT closely approximates BPTT, while exclusively using information that is locally available at the synapse.

### RECOLLECT selectively gates relevant information into working memory

**Fig 3:**
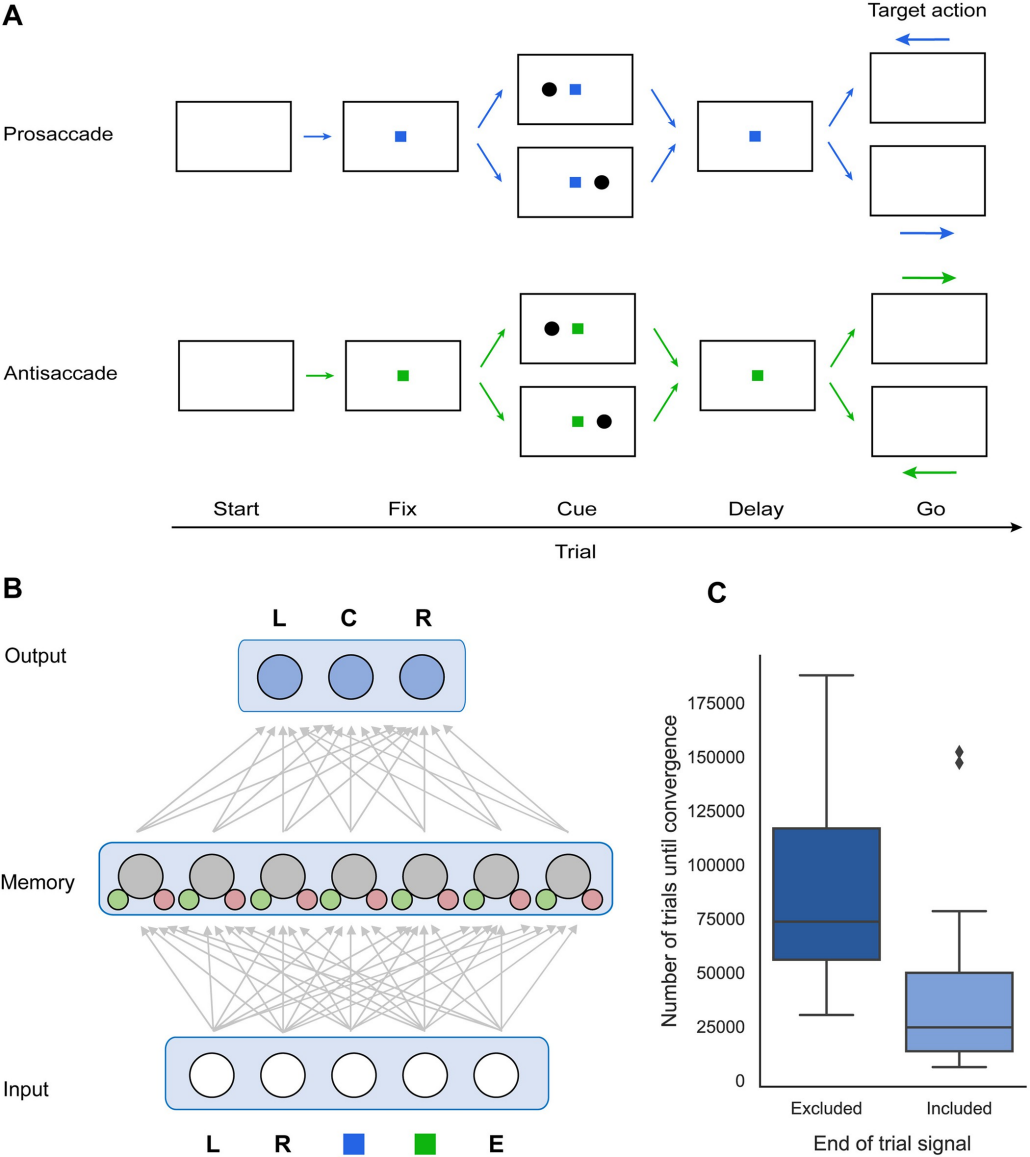
Structure of and performance of RECOLLECT on the pro-/anti-saccade task. A) Structure of the pro-/anti-saccade task. The fixation colour indicates whether a pro-saccade (blue) or anti-saccade (green) to a cue on the left or right side of the fixation mark has to be performed after a memory delay. B) Schematic representation of network architecture. The input layer in RECOLLECT receives information about the colour of the fixation marker (blue and green squares) and the position of the cue (L = left, R = right). An optional fifth input unit encoded the case end-of-episode signal (E). The output layer encodes the three actions that can be taken: Gaze directed to the left (L), centre (C) or right (R). C) Number of trials before convergence without an end of episode signal (left) or when it is included in the input (right). Boxes represent the first and third quartiles, with the middle line indicating the median. The whiskers range from the first quartile minus 1.5 times the interquartile range to the third quartile plus 1.5 the interquartile range. Outliers are indicated with diamonds.

Our goal was to develop a model that can learn to memorise and forget using a local, biologically plausible learning rule. To investigate how RECOLLECT gates information into its working memory and how it sustains these memory representations over time, the model was trained on the pro-/anti-saccade task from Gottlieb and Goldberg [36] (Fig 3A). This task was previously used to train AuGMEnT [16], which also used a biologically plausible learning rule but could not forget. Hence, the task is useful to illustrate differences between these models. The task consists of 50% pro-saccade trials in which the model should make a saccadic eye movement to a cued location after a memory delay and 50% anti-saccade trials in which the eye movement must be made in the direction opposite to where the cue appeared.

The model could direct gaze to the centre of the screen or to a position on the left or the right of the screen, by activating a corresponding unit in the output layer (Fig 3B). The task started with an empty visual display, after which either a blue or green fixation marker appeared in the centre of the screen (Fig 3A). A blue fixation marker signalled that a pro-saccade would be required and a green fixation marker an anti-saccade. If gaze was not directed to the centre position within 10 timesteps upon presentation of the central cue, the trial was terminated without reward. Otherwise, the model received a reward of 0.2 arbitrary units and was presented with a cue on either the left or the right side of the fixation marker during a single timestep. Once the cue disappeared, a memory delay of 2 timesteps commenced. If gaze fixation was broken before the end of this delay, the trial was aborted without additional reward. If the model kept fixating, the central fixation marker disappeared and the model had to make the appropriate saccade within 8 timesteps to receive a reward of 1.5 arbitrary units. There was an inter-trial interval of one timestep before the next trial started.

Hence, correct performance depended on the saccade direction which was determined by a non-linear combination of the colour of the fixation point and the cue location, which had to be memorised, requiring the maintenance of information until the ‘go’ cue. To prevent interference, the model should forget the cue location before the memory epoch of the successive trial.

There were two input units coding for the possible colours of the fixation marker (one-hot encoding) and two input units for the left or right cue (Fig 3B). The network was trained for a maximum of 1.000.000 trials or until convergence. Convergence was established if 1) the model had reached criterion performance (85% correct trials) on the last 100 trials of the four trial types (i.e. pro-saccade left, pro-saccade right, anti-saccade left and anti-saccade right), and 2) when it could perfectly complete all four trial types with its weights fixed and without exploration (i.e. learning was disabled).

We trained 20 networks with 4 input units, 7 memory units and 3 output units (Fig 3B) and randomly initialised, fully-connected weights. All networks reached the convergence criterion, indicating that RECOLLECT indeed successfully utilised its working memory. However, more training was required before convergence than in the previous AuGMEnT model although the network size was comparable (see Methods). Specifically, the median number of trials required was 73,614 for RECOLLECT, but only 4,100 for AuGMEnT. We note, however, that there are important differences between RECOLLECT and AuGMEnT. Memory units of AuGMEnT are perfect integrators and their activity is reset at the end of every trial. In contrast, RECOLLECT needs to learn to maintain information during a trial by the appropriate setting of the memory gates, and to later forget before the memory epoch of the successive trial. Hence, RECOLLECT learns about the structure of the environment, how it is composed of trials, as well as when and what to memorise. The comparison with AuGMEnT reveals that its versatile gating mechanism requires additional training time.

We hypothesised that learning with RECOLLECT could accelerate if we would add an explicit cue indicating the termination of a trial, since the network might learn to flush its memory upon receiving this signal, improving the learning process. Indeed, the inclusion of this end-of-trial signal reduced the median number of trials before convergence from 73,614 to 24,657 trials (*Z* = -2.99, *p* = 0.003, Wilcoxon signed-ranks test, for 20 randomly initialised networks with and without reset signal) (Fig 3C).

To investigate how RECOLLECT solves the pro-/anti-saccade task, we examined the activity profile and tuning of the units. In this analysis, we first increased the memory delay to five timesteps and the intertrial interval to three timesteps, using a curriculum (Materials & Methods).

**Fig 4:**
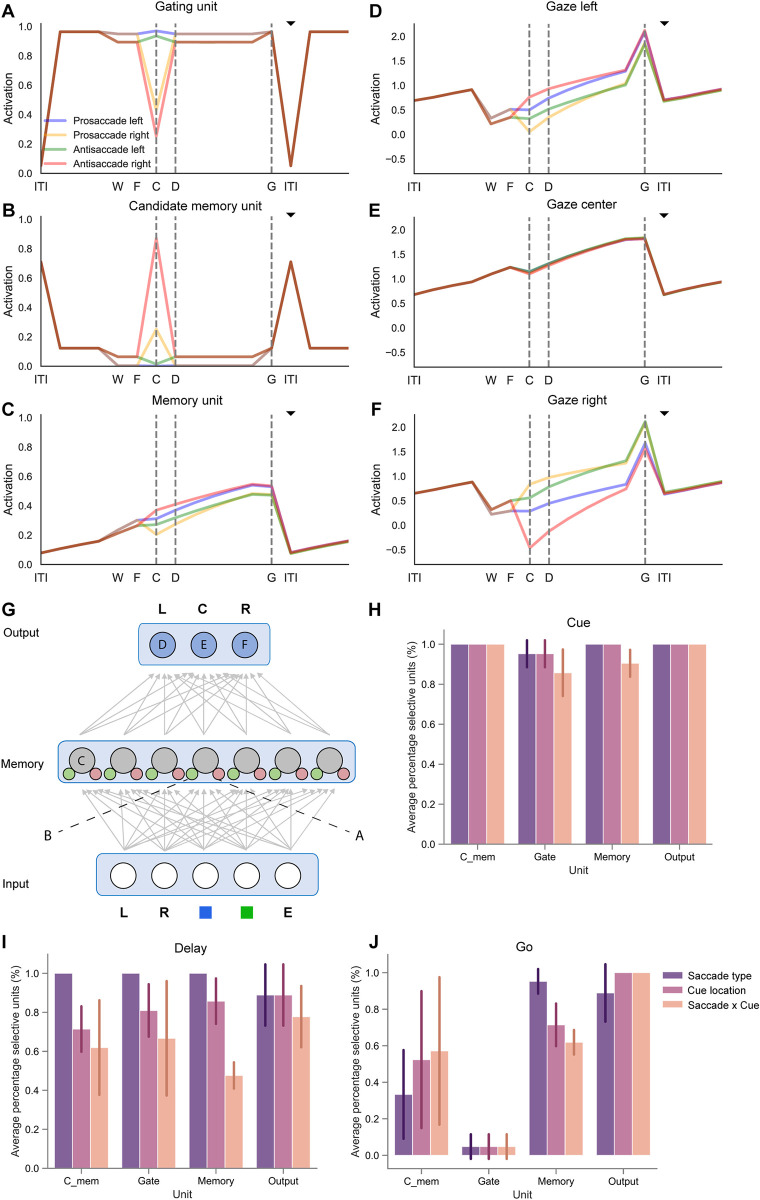
The selectivity and activity of units in networks trained on the pro-/anti-saccade task. A-F) The activity of example units on pro/anti-saccade trials with a left or right cue are shown in different colours. Pro-saccade trials with a cue on the left (right) are shown in blue (yellow) and anti-saccade trials with a cue on the left (right) in green (red). The black triangle indicates the time step when the end-of-trial signal was given. A) Example of a gating unit that was sensitive for cues on the left side, with strong activity on pro-saccade trials. Note the weak activity during the end-of-trial signal, which causes forgetting. B) A candidate memory unit that responded to right cues on anti-saccade trials. C) A memory unit that prefers trials with leftward saccades. D-F) The output units estimated the Q-value of a leftward saccade (D), fixation (E) and a rightward saccade (F). G) Architecture of RECOLLECT models trained on the pro-/anti-saccade task with labels referring to the example units from one network plotted in panels E-J to illustrate how RECOLLECT solves the pro-/anti-saccade task. H-J) Average percentage (+/- s.d.) of units selective for saccade type (pro- or anti-saccade), cue location (left or right), and their interaction, across three initialisations of the network. During cue presentation (H), nearly all units are selective for multiple features. During the delay (I), most units are selective for saccade type and a majority is also selective for cue location and the interaction between these factors. During the ‘go’ epoch (J), only few gating units exhibit selectivity. The selectivity of candidate memory units varies, whereas most memory and output units are selective for both features and their interaction. Labels: ITI = intertrial interval, W = waiting period until fixation is acquired, F = time of fixation, C = cue presentation, D = memory delay period onset, G = go-signal, i.e. the disappearance of the fixation point.

Units developed selectivity for the type of saccade (pro- or anti-saccade), the location of the visual cue (left or right), and to combine these two types of information to select the appropriate saccade. To investigate how this information can be combined across units to solve the task, we plotted the activity of example units in one of the networks (Fig 4G) for the four trial types (Fig 4A–4F). For instance, the gating unit illustrated in Fig 4A responded to left cues and was slightly more active on pro-saccade trials. In general, gating units often showed high activity for a particular feature (e.g. the blue marker, cueing pro-saccades) to facilitate memory while causing forgetting for the opposite feature (e.g. the green marker, cueing anti-saccades). Some units were selective for only one of the four trial types, such as the candidate memory unit in Fig 4B, which was most active for anti-saccade trials with a cue on the right. Several memory units developed selectivity for the required saccade direction, coding for the appropriate eye movement during the memory delay on both pro- and anti-saccadic trials. For instance, the memory unit in Fig 4C displayed a selectivity for leftward eye movements. As required by the task, the output unit with the highest Q-value was the one coding for the required action. Small differences between the Q-values suffice for convergence, because the network usually selects the action with the highest Q-value. The Q-values for the erroneous actions should eventually evolve to zero if training would continue. Finally, several units coded for the end-of-trial signal (Fig 4D–4F) so that the network flushed the memories to prevent interference on subsequent trials.

Fig 4H–4J shows the percentage of units that exhibited significant selectivity for these features and their interaction, across three initialisations of the network shown in Fig 4G. As can be observed, most units–irrespective of unit type–were significantly selective for all task features during cue presentation (Fig 4H). More variability could be seen during the memory delay period (Fig 4I), but in general most units coded for saccade type, with a large number of units also showing selectivity for the cue location, as well as the interaction between cue location and saccade type. Diverging selectivity profiles between unit types primarily emerged during the ‘go’ phase (Fig 4J), wherein gating units exhibited nearly no tuning to task features and only a relatively small number of candidate memory units being selective for saccade type, cue location and their interaction. However, nearly all output units and the majority of memory units were selective for all task features during this phase.

**Fig 5:**
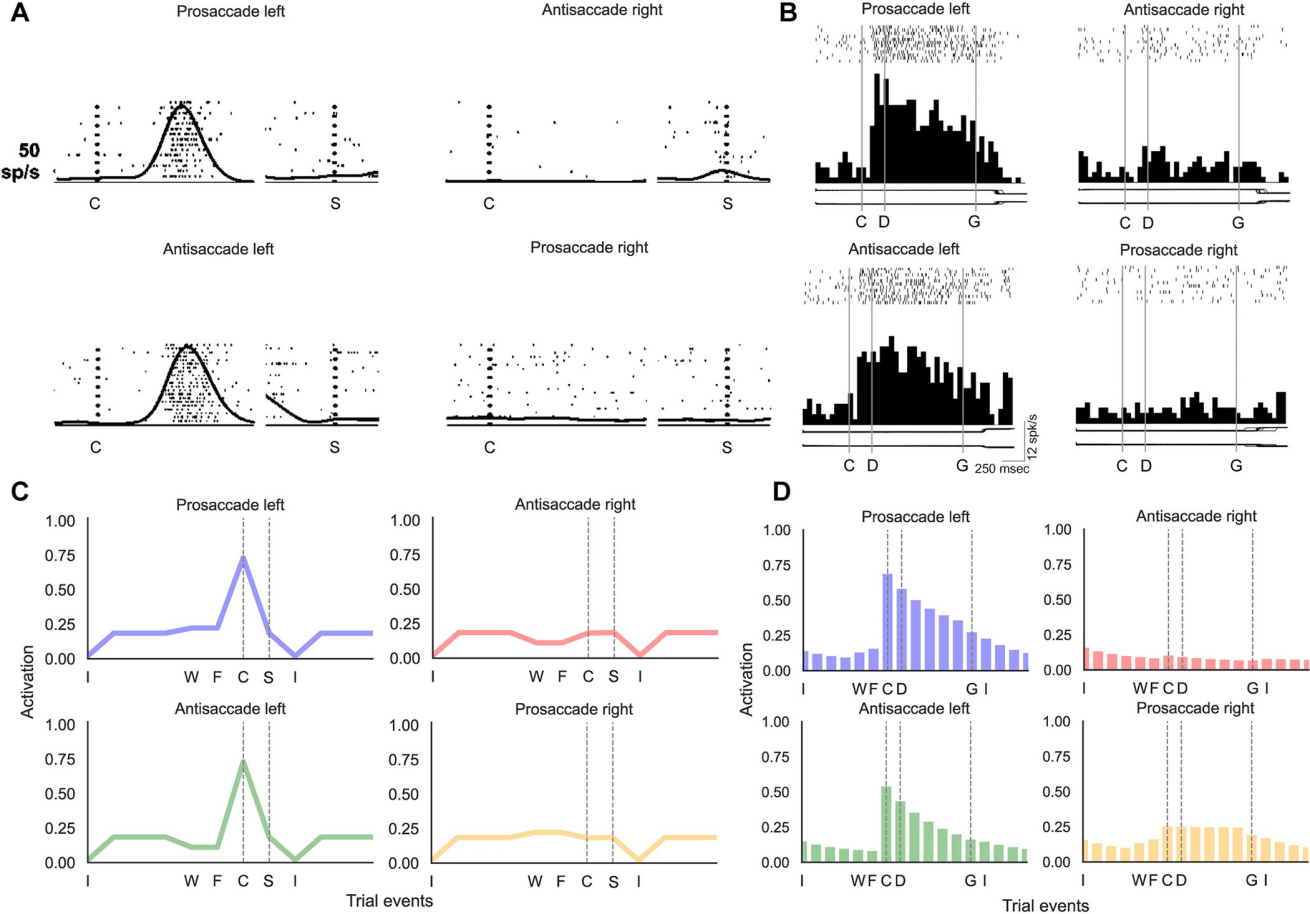
Comparison between neuronal data recorded in the parietal cortex of monkeys and RECOLLECT on the pro-/anti-saccade task. A) Example neuron in area LIP in the parietal cortex of a monkey coding for a visual cue on the left (adapted from Gottlieb & Goldberg [36]). The left and right dashed lines indicate cue and saccade onset, respectively. B) The activity of an example LIP memory cell for coding for cue location (adapted from Zhang & Barash [38]). Dashed lines signify cue onset, the memory delay period, and go-time (disappearance of the fixation cue, prompting saccade onset). C) Candidate memory unit in RECOLLECT coding for the left cue. D) Memory unit in RECOLLECT. Labels: I = intertrial interval, W = waiting period until fixation is acquired, F = time of fixation, C = cue presentation, D = memory delay period, G = go, S = saccade onset. Note that the conditions are ordered in the same way in panels C and D as the neurophysiological data in A and B, respectively.

Gottlieb and Goldberg [36] and Zhang and Barash [37,38] studied the selectivity of neurons in the lateral intraparietal area (LIP) in monkeys during a pro/anti-saccade task. Gottlieb and Goldberg [36] found that many neurons in a no-delay version of the task responded to one of the cues and did not show selectivity upon saccade onset (Fig 5A), whereas a smaller number of LIP neurons coded for the saccade direction. Zhang and Barash [38] used a memory delay, and reported a subset of neurons representing the memory of the cue location by firing persistently during the delay (Fig 5B). Yet other LIP neurons encoded the required motor response, or a non-linear combination of the stimulus position and the required eye movement. Units of networks trained with RECOLLECT expressed all these activity profiles (Fig 5C and 5D).

**Fig 6:**
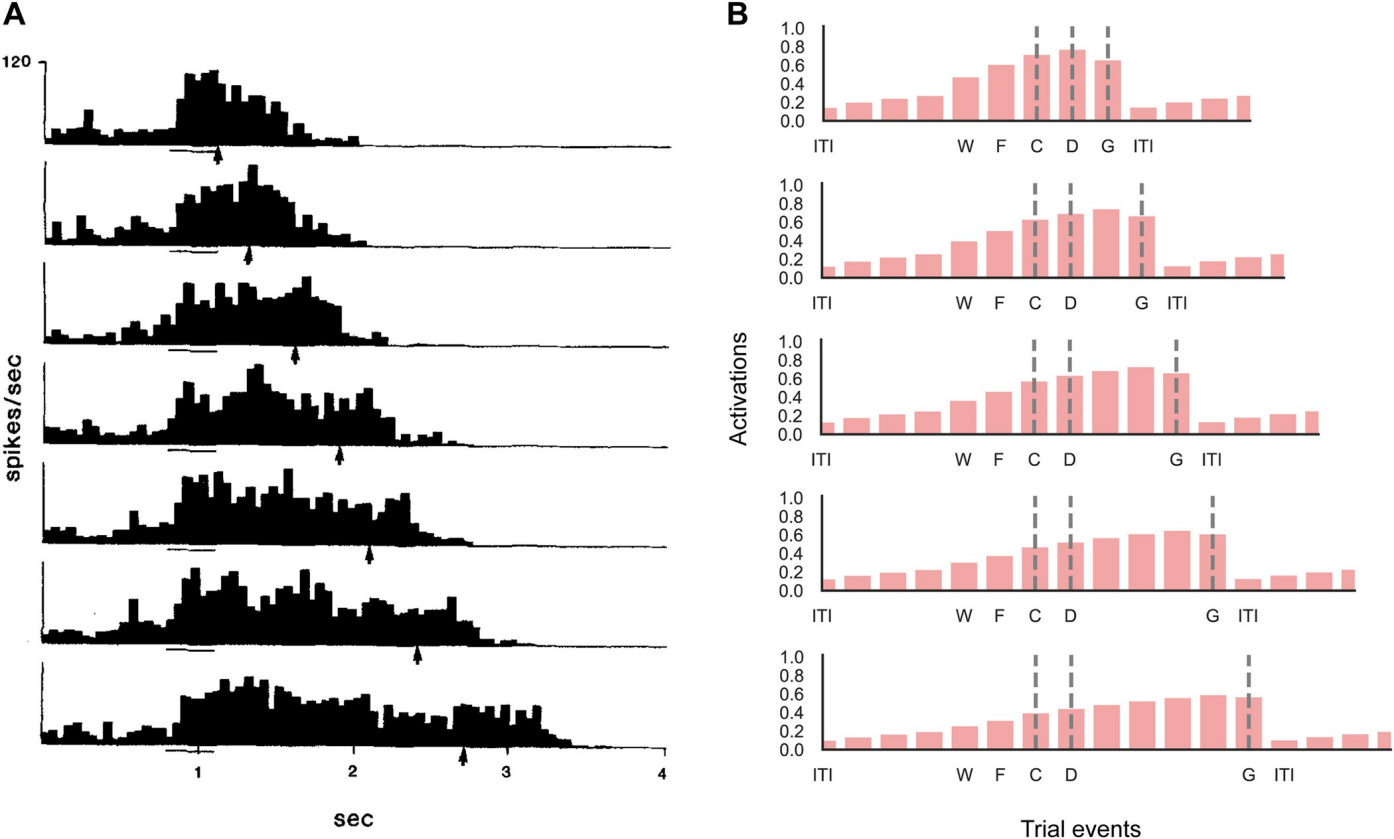
Sustained memory delay activity in the parietal cortex of monkeys and of RECOLLECT units on the pro-/anti-saccade task. A) Neurons in lateral interparietal cortex (LIP) in the parietal cortex of macaque monkeys persistently fire for the length of the memory delay of the pro-/anti-saccade task [39]. B) Memory units in RECOLLECT also exhibit persistent firing across increasingly long delays (1, 2, 3, 4 or 5 timesteps), which ceases when the memory epoch ends. Labels: ITI = intertrial interval, W = waiting period until fixation is acquired, F = time of fixation, C = cue presentation, D = memory delay period, G = go cue, which was cued by the disappearance of the central fixation point.

Other neurophysiological studies demonstrated that the duration of the persistent activity depends on the length of the period that the stimulus needs to be remembered. When the memory delay is extended the memory activity of LIP neurons persists longer [39] (Fig 6A). To investigate whether RECOLLECT displays a similar behaviour, we trained a network with varying memory delays (from one to five timesteps) (Fig 6B). The duration of persistent activity depended on the length of the delay, after which it declined upon the end-of-trial signal.

We conclude that RECOLLECT can train networks on the pro/anti saccade-task. These networks learn to memorize and forget when necessary and use persistent activity to code for memories in a similar manner as neurons in the brain.

### RECOLLECT exhibits learning-to-learn on a reversal bandit task

**Fig 7:**
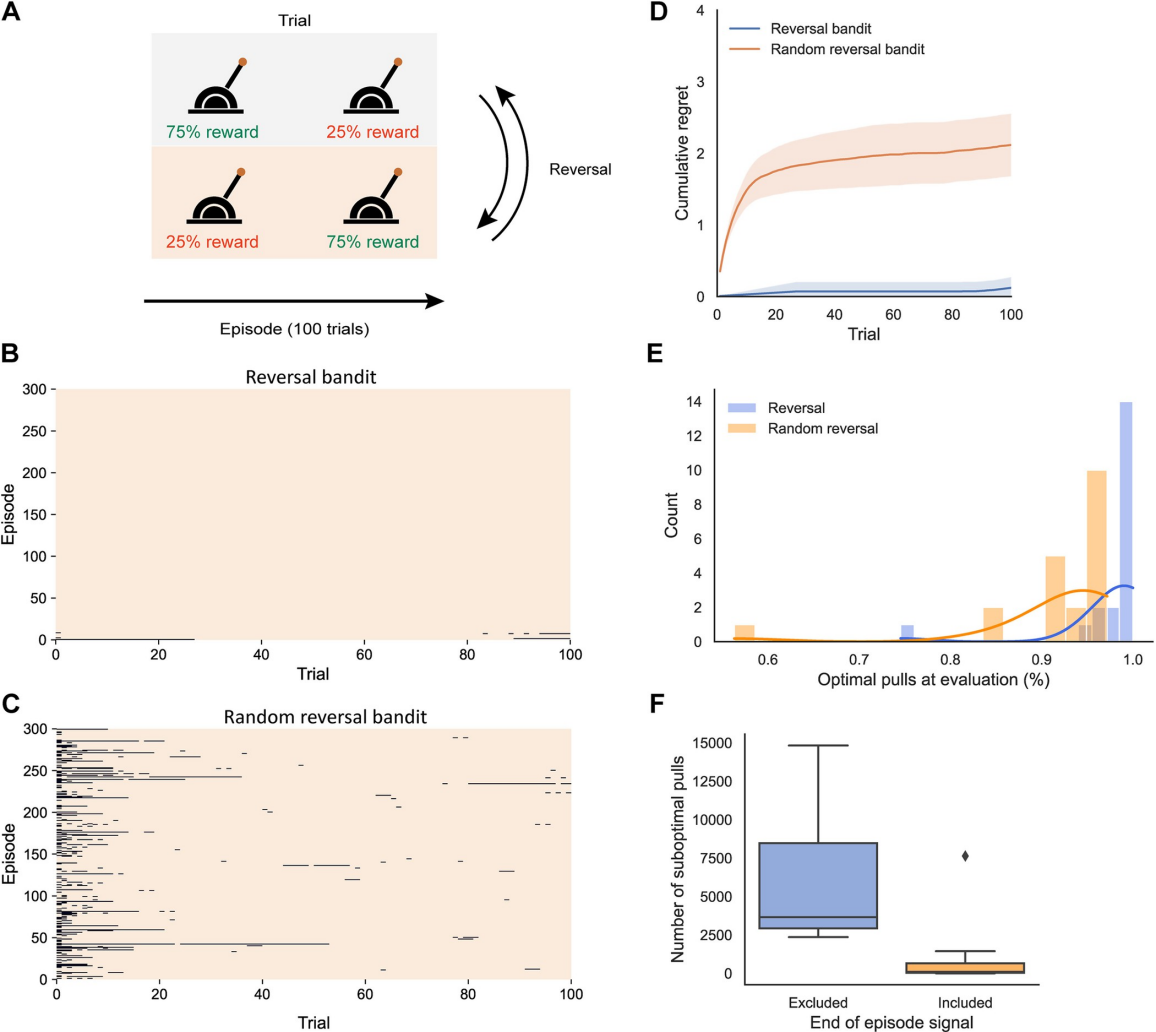
Structure of and performance of RECOLLECT on the reversal bandit task. A) Two-armed bandit reversal task. In the random version, we randomly assigned reward probabilities to the two levers when a new episode started. B,C) Performance on example networks after training on the reversal bandit (B) and random reversal bandit (C) at evaluation (99.8% and 97.2% optimal pulls, respectively). The networks were initialized with the same seeds. Orange and black regions denote optimal and suboptimal choices, respectively. Trials are shown on the x-axis, with 100 trials per episode, and successive episodes on the y-axis. D) Cumulative regret (± 95% confidence interval) on the reversal bandit task (blue) and the random version (orange). E) Histogram of the percentage optimal pulls on evaluation trials of the reversal bandit and random reversal bandit for the same 20 random seeds. F) The number of suboptimal pulls (300,000 pulls in total) in the non-random reversal bandit task is lower when an end of episode signal is included, cueing the model that the reward contingencies reverse.

We next investigated whether RECOLLECT can be used to train networks to learn-to-learn on a reversal bandit task (see Fig 7A). This task has previously been used to assess meta-learning (e.g. Wang et al. [9]) because its overarching reward structure can be learned and exploited.

On each trial during the task, the model chooses between two levers, of which one has a high (75%) reward probability and the other has a low (25%) reward probability. The task consisted of two contexts because the reward probabilities could reverse. Episodes consisted of 100 lever pulls and after every episode the reward probabilities were either reversed (reversal bandit), or randomly reassigned (random reversal bandit). The network had to sample the levers to assess the context, i.e. determine which one yielded the higher reward and then harvest rewards by consistently pulling this lever until the end of an episode. The reversal bandit is easier than the random reversal bandit because the network can exploit the predictable reversal between successive episodes.

Successful meta-learning on this task implies that a trained model can quickly (i.e. within one or just a few trials) switch to the new context at the start of a new episode by associating each context with a memory state. The model should change strategy when the preferred lever starts giving less reward, but the model needs to integrate information across several trials in which reward is unexpectedly omitted, because the best choice is only rewarded on 75% of the trials. The model could learn to use its working memory to represent the context by integrating information about the reward probability of the levers, as opposed to the much slower solution of relearning its weight structure upon every switch in the context. To facilitate meta-learning, the network had access to the action that it took on the previous timestep and the reward it received, which is informative about the current context. We also provided a signal that an episode had ended.

We trained RECOLLECT with 4 input units, 4 gating, candidate memory and memory units each (5 for the random reversal bandit). The two output units represented the two lever actions. We presented 20,000 episodes of 100 trials each (as in [9]). Once the training phase was completed, learning and exploration were disabled and the model completed an additional 300 evaluation episodes. We evaluated performance as the number of choices of the low-rewarding (i.e. suboptimal) lever on 20 random initialisations of the network. For comparison with Wang et al. [9,40], we also provide a measure of cumulative regret. Regret occurs when the action taken deviates from the optimal action (under hindsight) and a reward is not obtained. Cumulative regret refers to the cumulative loss of these expected rewards over time [41].

Fig 7B and 7C illustrate the suboptimal pulls as black line segments during the evaluation phase of two networks that were trained on the reversal and random reversal bandit tasks, respectively. The example network trained on the reversal bandit task learned to select the correct lever upon episode reversals almost perfectly. Suboptimal pulls only occurred either at the beginning of an episode or just before the end. There were more suboptimal arm pulls on the random reversal task, which were concentrated at the beginning of episodes. While RECOLLECT tended to select the correct lever thereafter, there were also some episodes with errors at other time points. We predicted that these occurrences might occur due to the absence of an expected reward on several consecutive trials, thereby falsely suggesting a context switch. In accordance with this view, the average reward received on the previous three trials was 0.74 when a correct response was made but only 0.23 when incorrect choices were made.

Networks trained on the reversal bandit task (see Fig 7E) achieved a median accuracy of 99.7%, with some networks reaching 100% optimal pulls. As expected, the accuracy on the random reversal bandit was significantly lower at 94.9% (Wilcoxon Signed-Ranks test, *z* = -3.06, *p* = .002). Hence, RECOLLECT exploited the regularity of the reversal bandit task, in which the episodes always alternated and the network did not have the sample the new reward structure when a new episode started. The performance of RECOLLECT on the random reversal bandit (see Fig 7D) was only slightly below that of long-short term memory (LSTM)-based architectures trained in the same learning-to-learn setting, with an average cumulative regret of 2.1 for RECOLLECT (97.2% optimal pulls) versus 1.1 in Wang et al. ([40]; 98.5% optimal pulls). This is remarkable, given the reduced computational complexity of RECOLLECT and its use of a local, biologically plausible learning rule.

To investigate the effect of the end-of-episode signal, we trained 20 networks with and without this signal on the non-random reversal bandit (Fig 7F). At evaluation, the median number of suboptimal pulls of these networks was 99 (of a total of 300,000 pulls) in the presence of the end-of-episode signal, which was significantly lower than the median number of 3,661 suboptimal pulls without this signal (Wilcoxon signed-ranks test, *Z* = -3.47, *p* < .001). Hence, RECOLLECT capitalises on the end-of-episode signal to increase its performance.

**Fig 8:**
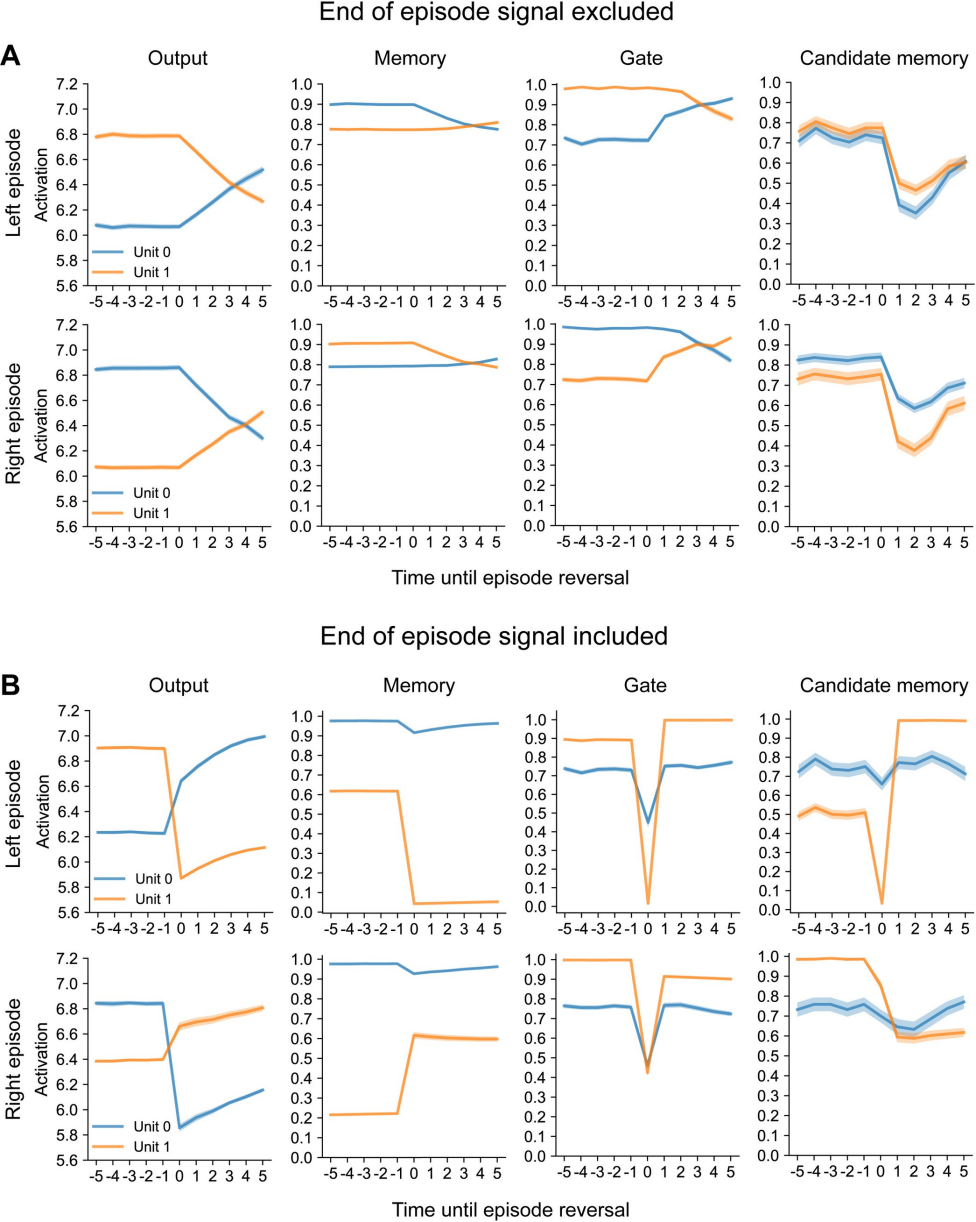
The average of example units in the reversal bandit task. 8. (A) Example network trained in the absence of an end-of-episode signal. The activities of the two Q-value units in the output layer reverse after the episode. The network has to gather evidence across trials in its memory units (second column) based on the reward contingency that the context changed, because only 75% of the optimal choices are rewarded. The third and fourth columns show the activity of the gating and candidate memory cells, respectively. The shading shows ± *SEM*. (B) The reversal of the state of the network is abrupt in the presence of the end-of-episode signal, which changes the memory state of the network within one trial.

We analysed a smaller network, with only two memory units, to gain insight into how it solves the reversal bandit task. We plotted the average activity (± *SEM*) across episodes of network units for left and right high-rewarding episodes before and after reversals for an example network (Fig 8). We will first discuss activity in the absence of an end of episode signal (Fig 8A). Before the reversal, the activity of the Q-value unit coding for the highly rewarded action was higher than that of the other Q-value unit. This pattern reversed slowly after the switch (*t* = 0) until the unit for the now appropriate action was more active (around 4 trials after the reversal). This strategy reflects the accumulation of evidence for a switch in context. Because the correct lever is only rewarded 75% of the time and the incorrect lever yields a reward on 25% of the trials, a single rewarded or unrewarded lever pull does not give reliable information about the context. Instead, RECOLLECT needs to integrate outcome information across a few trials until it can determine that the context changed. Note that the Q-values exceed the reward value the network can receive on a single trial. Instead, these values reflect the discounted reward expectation across a number of trials given that a particular action is chosen.

The activity of Q-value units depended on the activity of memory and gating units, which had comparable activity time courses. Interestingly, the activity of one of the gating units was close to one until the reversal, which indicates that the memory was maintained (Fig 8A). When the episode ended, the activity of the gating unit decreased, permitting an influence of the candidate memory units and the reversal of activity of the gating and memory units.

The activities of the two candidate memory units indicated selectivity for the context (Fig 8A). Their activity decreased upon the absence of expected reward due to the change in context, followed by a slower recovery. Hence, the network learned to represent the task context in its working memory by integrating information across chosen actions and obtained rewards across a number of successive trials, in the absence of explicit reset signals.

The activity of the network that was trained with an end of episode signal was drastically different (Fig 8B). The switch in the activity of Q-value units occurred within a single trial, indicating that the network learned the significance of the end-of-episode signal and efficiently changed its working memory to select the correct, alternative lever in the successive episode. In the example network, one of the memory units exhibited sharp decreases and increases upon episode reversals for left and right episodes, respectively (orange in Fig 8B), driving the change in the Q-values in the output layer. Both gating units exhibited steep declines in activity in response to the end-of-trial signal. Finally, one of the candidate memory units (orange in Fig 8B) was very active during right high-rewarding episodes, and less during left high-rewarding episodes. In summary, RECOLLECT rapidly switched between memory states in the presence of an end of episode signal, which improved the efficiency on the reversal bandit task.

**Fig 9:**
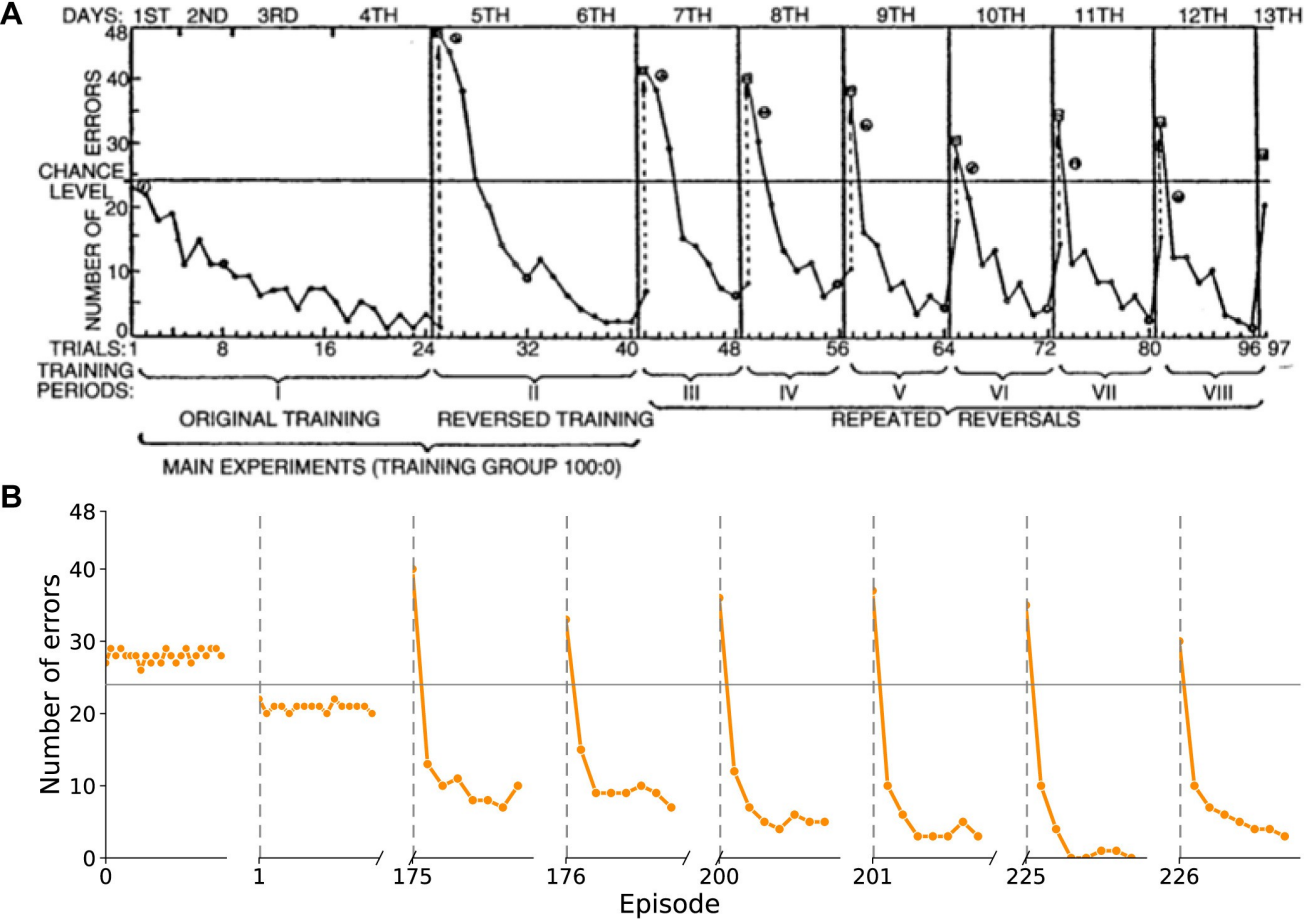
Performance in a reversal task of rats and of networks trained with RECOLLECT. (A) Learning decreases the number of errors that rats make on a reversal bandit task (data from [42]). Y-axis, number of rats (total 48) making an error. (B) Networks trained with RECOLLECT. Each data point represents the number of errors per trial in successive episodes, summed across 48 networks. We plotted the first 24 trials of the first episode, 16 trials after the first reversal and 8 trials of subsequent episodes.

Finally, we compared the behaviour of networks trained with RECOLLECT on the non-random reversal bandit task to the choices made by rats trained on a similar task. Brunswik [42] trained rats on serial-reversal task on a T-maze, with two arms that were baited with different rewards. On the first 24 trials, one arm was always rewarded and the other arm was never rewarded. Rewards were reversed for the subsequent 16 trials. This was followed by several reversal episodes of 8 trials each, until the rats completed a total of 8 episodes. During the first episode the performance gradually increased (Fig 9A). The first reversal caused a sharp increase in errors, which then declined, a pattern that repeated for every reversal afterwards. Interestingly, the rats required fewer trials to accommodate the later switches, indicating that the rats learned-to-learn this task.

We next analysed the appearance of switching behaviour for 48 networks trained with RECOLLECT (Fig 9B), baiting the highly rewarding lever on 100% of trials with a reward and the other lever on 0% of the trials. Learning in RECOLLECT is slower than that of rats, and we therefore plotted the number of errors in the first episode, the first reversal and the 175^th^, 200^th^ and 225^th^ episodes with the subsequent reversals (episode 176, 201 and 226). This difference in learning rate is presumably due to the fact that RECOLLECT is initialised *tabula rasa* at time of training, unlike the rats. The evolution of behaviour in RECOLLECT, however, was similar to that of the rats. Episodes started with many errors, after which the accuracy improved in later episodes, similar to what was observed by Brunswik et al. [42].

In conclusion, RECOLLECT can successfully train networks on the reversal bandit task in a way that is comparable to non-biologically plausible models. Moreover, the progression of learning is qualitatively similar to the behaviour of rats in a reversal task.

## Discussion

We developed a novel gated memory network that could memorise task-relevant information, forget it when appropriate and learned-to-learn in a biologically plausible manner. The model incorporated a version of the light-gated recurrent unit (Light-GRU [12]) and its learning rule was based on AuGMEnT [16] that uses a combination of attentional feedback and neuromodulators that code for the RPE. The result is a biologically plausible form of learning that is similar to backpropagation-through-time. In RECOLLECT, all information used to update the network is locally available at the synapses of the network. Specifically, candidate memory, gating and memory units could be considered part of the same cortical column or loop through subcortical structures and the attentional feedback signal as a locally available signal in that column. Indeed, neurons in the different layers of the cortex play specific roles in representing sensory input, attention and working memory [43]. Hence, RECOLLECT provides a biologically plausible learning rule for gated memory networks, which differentiates it from AuGMEnT, which required a reset of its memory after every trial.

The main advantage of the RECOLLECT architecture with memory gates is its flexibility. Whereas its predecessor AuGMEnT remembers by default and cannot learn to forget [16], RECOLLECT learns to strategically flush its memory when useful. The Light-GRU is one of the simplest memory units with this property [12], making it a useful component of neuronally plausible models to study the mechanisms underlying memory and forgetting compared to larger and LSTM-based networks, which are more difficult to interpret [9]. We note, however, that the precise mapping of the gating mechanisms onto the circuits underlying memory and forgetting in the brain remains to be elucidated. Previous neuroscientific studies revealed multiregional loops between the cortex, thalamus and striatum for working memory [44–47]. Recent evidence also points towards a role of the loop through the cerebellum in working memory [48–51]. These loops have also been implied in reversal learning [52,53]. More research is needed to fully comprehend how these circuits effectuate working memory and forgetting. The learning rule for the gating connections compared the memory and the new input (Eq 14). This comparison plays a prominent role in theories of predictive coding [31], thereby establishing a new link between theories of predictive coding and biologically plausible learning rules.

We tested RECOLLECT on a pro-/anti-saccade task, and found that the model flexibly selects which information to remember during a delay. Moreover, RECOLLECT learned to flush its memory at the end of a trial to prevent interference of the memories on subsequent trials, representing an improvement over the AuGMEnT model. A comparison of units in networks trained with RECOLLECT to neurophysiological data revealed many similarities. Units developed selectivity for the colour of the fixation marker and the position of the cue, as well as persistent firing coding for the relevant features, just as been observed in the visual and parietal cortex of monkeys [36,38,39]. Thus, RECOLLECT is not only biologically plausible given its reliance on neuromodulators and attentional feedback signals, but networks trained with RECOLLECT develop units that resemble neurons in the brains of animals that have learned the same tasks.

We used a reversal bandit task to test whether RECOLLECT learned-to-learn. Networks trained with RECOLLECT sampled the environment to gauge which of the two levers yielded the highest reward, and it then consistently chose this lever until the end of the episode. Moreover, the model’s behaviour during learning was reminiscent of how rats learn the reversal bandit task [42]. There was an initial increase in errors upon the start of a new episode that decreased over the course of the episode. These errors declined more quickly as training progressed, indicating a similar progression of learning-to-learn in the model and in rats.

An interesting observation pertained to the role of the end-of-trial signal in the pro-/anti-saccade task and the end-of-episode signal in the reversal bandit task. These signals enhanced performance by providing a signal that it is time to update the memory state; thereby simplifying the problem, because the network did not have to integrate information about the relation between stimulus, response and reward to detect a reversal. Likewise, cells in the prefrontal cortex have been shown to represent action sequence boundaries by increased firing rates following the end of the sequence [54]. We found that RECOLLECT networks took advantage of cues signalling a reversal, by rapidly switching to the new strategy. The network also learned to integrate information about the rewards across a number of trials, when the change in the reward contingencies was not signalled explicitly. Hence, RECOLLECT parallels aspects of animal learning such as the identification of sequence boundaries. The accumulation of evidence across trials for a switch of context resembles the activity of neurons in the anterior cingulate cortex of monkeys, which also accumulate evidence based on delivered rewards that the context might have changed [55,56].

We here only tested RECOLLECT with a single layer with memory units. Future work could expand RECOLLECT for more complex tasks with multiple memory layers for simple and more complex features. Furthermore, while RECOLLECT consistently converged on the pro-/anti-saccade task, learning was slower than with the previous AuGMEnT architecture [16], which remembers by default. Similarly, RECOLLECT performed slightly less well on the random reversal bandit than LSTM-based networks trained in the same learning-to-learn setting. These differences are partially explained by the extra information that was given to the previous models. For example, in the study on AuGMEnT [16] and in Wang et al. [9], the network state was reset at the end of each trial. In a variant of AuGMEnT that had to learn to reset its working memory itself, learning was slower than in standard AuGMEnT [57]. RECOLLECT stands out because it learned the time structure of the task, what to remember and when to forget it. The network took advantage of end-of-trial signals, but learning was even possible when such a signal was not presented.

We implemented a few modifications to the Light-GRU units [12]. The main change is that we excluded recurrent weights from memory units to other memory and gating units. This modification allowed the correspondence to BPTT (see Eqs 9–16) in a simpler model. Such simplicity sometimes enhances performance [13,58] and RECOLLECT learned the tasks that we studied here without these additional connections. Nevertheless, the RECOLLECT learning rule is compatible with architectures in which these connections are present and future studies could include them, because they might benefit learning of more complex tasks.

There are other learning rules and models that approximate backpropagation-through-time (e.g. [17,59]. RECOLLECT uses the same approximation as AuGMEnT and also e-prop [17], which has been used to train long-short term memory models in reinforcement learning settings. There are a number of important differences between RECOLLECT and e-prop. Firstly, RECOLLECT incorporates synaptic tags that implement the faster TD(*λ*) algorithm, rather than the simpler TD(0) method [60]. Secondly, e-prop requires each unit to be connected to an output unit to propagate the error signal. Hence e-prop cannot train the lower layers of deeper networks, effectively limiting the approach to shallow networks. In contrast, RECOLLECT can be extended to deeper networks, just like AuGMEnT [16] and BrainProp [61], and hence to more complex tasks. Thirdly, RECOLLECT uses the Light-GRU unit, which is much simpler than the long-short term memory units that were used by Bellec et al. [17]. There are also studies investigating learning-to-learn in spiking architectures [62–66], but we note that these still rely on BPTT for training or are less straightforward to implement in the brain because they use second-order gradients in the outer loop training process (i.e. the overarching learning problem where knowledge is accumulated over multiple learning experiences rather than just in a single trial), rather than the more biologically plausible meta-reinforcement learning method formalised by [9,10]. Finally, the previous ‘WorkMATe’ model [67] also used the AuGMEnT learning rule in a model for working memory. The mechanisms for memory and forgetting differ substantially between WorkMATe and RECOLLECT. WorkMATe relies on complex gated memory stores for sensory stimuli, which are updated in an all-or-nothing manner. A separate output module chooses whether new stimuli are encoded in one of the memory store blocks or forgotten. Hence, stored stimuli override previous memory content in WorkMATe, making memorizing and forgetting less flexible than in RECOLLECT.

In conclusion, RECOLLECT is a novel gated neural network that only uses information that is available locally at the synapse to learn how to use its working memory flexibly and learn-to-learn in a manner that is reminiscent to animal learning. It presents a biologically plausible alternative to more traditional gated memory networks such as long-short term memory. RECOLLECT thereby contributes to our understanding of how working memory, forgetting and learning-to-learn are implemented by the brain.

## Materials & methods

### Architecture details

#### Activation function

A sigmoid activation function determined the activity of gating units and candidate memory units:

$$\sigma ({input}_{j}(t))=\frac{1}{\left(1+\exp\left(-(\rho ∙{input}_{j}(t))\right)\right)}.$$ (M1)

where *ρ* represents the slope of the sigmoid. The value of *ρ* was set to 2 in all experiments.

#### Learning rate

The learning rate is shown in Table 1. We noticed that rapid plasticity of gating units decreased the stability of learning. We therefore set the learning rate of synapse onto gating units at a lower value than those of other connections.

**Table 1:** RECOLLECT hyperparameters for each task (variant).

	Pro-/anti-saccade task	Reversal bandit	Random reversal bandit
Exploration rate (ε)	0.025	0.025	0.025
Number of input units (including end of trial/episode signal)	5	4	4
Number of Light-GRU units	7	4	5
Number of output units	3	2	2
Learning rate (*β*)	0.1	0.01	0.005
Learning rate of gating units (*β*_*gate*_)	0.006	0.006	0.0005
Discount factor (γ)	0.9	0.9	0.9
Tag decay rate (λ)	0.4	0.2	0.1

#### Network parameters

During the initialisation, all biases (i.e. for the gating units, candidate memory units and output units) were set to one. For the other parameters, a grid search with a limited set of a priori chosen values was conducted for parameter optimisation. For this, the standard learning rate for all units (*β*), the learning rate specific to the gating units (*β*_*gate*_) and the tag decay rate (λ) were particularly important. Learning benefitted from lower values for these hyper-parameters in the bandit paradigms (especially the random reversal bandit), because of the more conservative updates in times of uncertainty and preventing premature decisions for a lever before sufficient information has been gathered. Unless otherwise indicated, the parameters used for the experiments were as follows:

#### Pro-/anti-saccade task

To facilitate comparison with Rombouts et al. [16], simulations regarding performance (Fig 3) on the pro-/anti-saccade task were performed using an intertrial interval of 1 time step and a memory delay of 2 time steps. In further stimulations (except for Fig 6) we used an intertrial interval of 3 and memory delay of 5 timesteps so that the neural activations during memory delay and after the end of trial signal could be studied more closely. A curriculum was used to achieve these longer memory delays. Specifically, we started with a delay of 1 time step. After the model reached criterion performance (85% correct trials on the previous 100 trials of each trial type), the memory delay was set to 2 time steps and then to 4 time steps until the final memory delay of 5 time steps was reached. Networks contained 7 Light-GRU units, and each of them was composed of a gating, candidate memory and memory unit. However, for Fig 5C and 5D 12 Light-GRU units were included. AuGMEnT was trained with 3 regular hidden units, 4 memory hidden units, and special input units which were either following the input (*N* = 4, instantaneous input units) or responded to the on- and offset of stimuli (*N* = 8 transient input units [16]). The total number of trainable weights was 75 for AuGMEnT and 94 for RECOLLECT.

The no-delay variant of the pro-/anti-saccade task for Fig 3A was implemented by first showing the fixation marker (F), followed by the cue without fixation marker (C). The disappearance of the cue prompted the saccade (S).

#### Reversal bandit

In order to understand how RECOLLECT solves the reversal bandit, the activation plots and neural data comparison figure were created with small networks with two gating, candidate memory and memory cells (Table 1).

To analyse the average reward on the previous three trials across episodes for the data in Fig 7C, only averages were calculated from the fourth trial onwards to prevent any confounding with episode reversal effects. To avoid biasing the analysis, only episodes with a mixture of correct and incorrect responses were included.

#### Statistical analyses

Prior to statistical analysis, assumptions of normality were tested using the Kolmogorov-Smirnov and Shapiro-Wilk tests. If these tests indicated significant deviations from normality for at least one of the two distributions, a non-parametric test was used and the median was reported instead of the mean.

We used a regression analysis to determine whether units showed significant selectivity to features in the pro-/anti-saccade task (Fig 6A–6C). We fitted a linear regression model with saccade type (pro-saccade or anti-saccade), cue location (left or right) and their interaction to the activity of units in three networks during the cue, memory delay or ‘go’ phases of the task. If an omnibus test for normality, Durbin-Watson or Jarque-Bera test, indicated significant heteroscedasticity, skewness or kurtosis (alpha of 0.05), a robust regression model was fitted using Huber’s t function instead. We included a Bonferroni correction for multiple comparisons and applied an alpha of 0.05.

### The relation between backpropagation-through-time and RECOLLECT

In this section, we will demonstrate that backpropagation-through-time is implemented by RECOLLECT with a combination of synaptic traces and tags.

#### Computing the gradient of M_j_(t), k_j_(t) and C_j_(t)

The influence of the activity of memory unit *j*, *M*_*j*_*(t)*, on the Q-value of the selected action *s*, *q*_*s*_*(t)*, is (Fig 1):

$$\frac{\partial q_{s}(t)}{\partial M_{j}(t)}=W_{sj}^{FB},$$ (M2)

which is proportional to the amount of attentional feedback flowing from the winning action *s* to memory unit *j* [68]. We can now compute the influence of the memory gate *k*_*j*_*(t)* on *q*_*s*_*(t)* based on Eq 3:

$$\frac{\partial q_{s}(t)}{\partial k_{j}(t)}=\frac{\partial M_{j}(t)}{\partial k_{j}(t)}\frac{\partial q_{s}(t)}{\partial M_{j}(t)}=[M_{j}(t-1)-C_{j}(t)]W_{sj}^{FB}.$$ (M3)

Furthermore, it follows from Eq 3 that the influence of *C*_*j*_*(t)* on *q*_*s*_*(t)* depends on *k*_*j*_*(t)*:

$$\frac{\partial q_{s}(t)}{\partial C_{j}(t)}=\frac{\partial M_{j}(t)}{\partial C_{j}(t)}\frac{\partial q_{s}(t)}{\partial M_{j}(t)}=[1-k_{j}(t)]W_{sj}^{FB}.$$ (M4)

### Computing the gradient of W^C^_ij_ using synaptic traces

We can now compute the *instantaneous* impact of connections *W*^*C*^_*ij*_*(t)* on *q*_*s*_*(t)*:

$$\frac{\partial q_{s}(t)}{\partial W(t)}=\frac{\partial C_{j}(t)}{\partial W_{ij}^{c}(t)}\frac{\partial q_{s}(t)}{\partial C_{j}(t)}=x_{i}(t)\sigma^{\prime }({Inp}_{j}^{C}(t))[1-k_{j}(t)]W_{sj}^{FB},$$ (M5)

where $\sigma \prime ({Inp}_{j}^{C})$ is the derivative of the activation function. However, these connections have also had impact on the memory state *M*_*j*_*(t)* on all previous time steps according to Eq (1). For example, connection *W*^*C*^_*ij*_ had an influence on *C*_*j*_*(t-1)* which influenced *M*_*j*_*(t-1)* and thereby also *M*_*j*_*(t)*. Although the notation is a bit ugly, for convenience let us write for this influence of *W*^*C*^_*ij*_ on *t-1* on *q*_*s*_*(t)*:

$$\frac{\partial q_{s}(t)}{\partial W_{ij}^{C}(t-1)}=\frac{\partial C_{j}(t-1)}{\partial W_{ij}^{C}(t-1)}\frac{\partial M_{j}(t-1)}{\partial C_{j}(t-1)}\frac{\partial M_{j}(t)}{\partial M_{j}(t-1)}\frac{\partial q_{s}(t)}{\partial M_{j}(t)}=x_{i}(t-1)\sigma^{\prime }({Inp}_{j}^{C}(t-1))[1-k_{j}(t-1)]k_{j}(t)W_{sj}^{FB}.$$ (M6)

We can also compute this term for *t-2*:

$$\frac{\partial q_{s}(t)}{\partial W_{ij}^{C}(t-2)}=\frac{\partial C_{j}(t-2)}{\partial W_{ij}^{c}(t-2)}\frac{\partial M_{j}(t-2)}{\partial C_{j}(t-2)}\frac{\partial M_{j}(t-1)}{\partial M_{j}(t-2)}\frac{\partial M_{j}(t)}{\partial M_{j}(t-1)}\frac{\partial q_{s}(t)}{\partial M_{j}(t)}=x_{i}(t-2)\sigma^{\prime }({Inp}_{j}^{C}(t-2))[1-k_{j}(t-2)]k_{j}(t-1)k_{j}(t)W_{sj}^{FB}$$ (M7)

and, in general, for *t-i*:

$$\frac{\partial q_{s}(t)}{\partial W_{ij}^{C}(t-i)}=x_{i}(t-i)\sigma^{\prime }({Inp}_{j}^{C}(t-i))[1-k_{j}(t-i)]\prod_{g=t-i+1}^{t}k_{j}(g)W_{sj}^{FB}.$$ (M8)

Although this gradient may look complex, it is actually straightforward to store the information in a *trace*^*C*^_*ij*_ at the synapse and update it based on information that is locally available:

$${Trace}_{ij}^{C}(0)=0,$$ (M9)

$${Trace}_{ij}^{C}(t)={k_{j}(t)Trace}_{ij}^{C}(t-1)+{[1-k_{j}(t)]x}_{i}(t)\sigma^{\prime }({Inp}_{j}^{C}(t)).$$ (M10)

Importantly, this information can be made available locally at the synapse, assuming that the gating unit *k*_*j*_ is in the same cortical column as the memory unit *M*_*j*_. Adding all the time steps, the total influence of *W*^*C*^_*ij*_ on *q*_*s*_*(t)* becomes:

$$\frac{\partial q_{s}(t)}{\partial W_{ij}^{C}}={Trace}_{ij}^{C}W_{sj}^{FB}.$$ (M11)

#### Computing the gradient of W^k^_ij_ using synaptic traces

We can use Eq (5) to compute the influence of the synapses *W*^*k*^_*ij*_ that influence the memory gate *k*_*j*_*(t)* on *q*_*s*_*(t)*. As before, we start with the *instantaneous* impact of connections *W*^*k*^_*ij*_*(t)* on *q*_*s*_*(t)*:

$$\frac{\partial q_{s}(t)}{\partial W_{ij}^{k}(t)}=\frac{\partial k_{j}(t)}{\partial W_{ij}^{k}(t)}\frac{\partial q_{s}(t)}{\partial k_{j}(t)}=x_{i}(t)\sigma^{\prime }({Inp}_{j}^{k}(t))[M_{j}(t-1)-C_{j}(t)]W_{sj}^{FB}.$$ (M12)

Let us now consider the influence of this synapse at *t-1* on the *q*_*s*_*(t)*:

$$\frac{\partial q_{s}(t)}{\partial W_{ij}^{k}(t-1)}=\frac{\partial k_{j}(t-1)}{\partial W_{ij}^{k}(t-1)}\frac{\partial M_{j}(t-1)}{\partial k_{j}(t-1)}\frac{\partial M_{j}(t)}{\partial M_{j}(t-1)}\frac{\partial q_{s}(t)}{\partial M_{j}(t)}=x_{i}(t-1)\sigma^{\prime }({Inp}_{j}^{k}(t-1))[M_{j}(t-2)-C_{j}(t-1)]k_{j}(t)\;W_{sj}^{FB},$$ (M13)

and at *t-2*

$$\frac{\partial q_{s}(t)}{\partial W_{ij}^{k}(t-2)}=\frac{\partial k_{j}(t-2)}{\partial W_{ij}^{k}(t-2)}\frac{\partial M_{j}(t-2)}{\partial k_{j}(t-2)}\frac{\partial M_{j}(t-1)}{\partial M_{j}(t-2)}\frac{\partial M_{j}(t)}{\partial M_{j}(t-1)}\frac{\partial q_{s}(t)}{\partial M_{j}(t)}=x_{i}(t-2)\sigma^{\prime }({Inp}_{j}^{k}(t-2))[M_{j}(t-3)-C_{j}(t-2)]k_{j}(t-1)\;k_{j}(t)\;W_{sj}^{FB}.$$ (M14)

In general, for *t-i*:

$$\frac{\partial q_{s}(t)}{\partial W_{ij}^{k}(t-i)}=x_{i}(t-i)\sigma^{\prime }({Inp}_{j}^{k}(t-i))[M_{j}(t-i-1)-C_{j}(t-i)]\prod_{g=t-i+1}^{t}k_{j}(g)\;W_{sj}^{FB}.$$ (M15)

This gradient can also be stored in the form of a *trace*^*k*^_*ij*_ at the synapse and updated based on information that is locally available:

$${Trace}_{ij}^{k}(0)=0,$$ (M16)

$${Trace}_{ij}^{k}(t)={k_{j}(t)Trace}_{ij}^{k}(t-1)+{[M_{j}(t-1)-C_{j}(t)]x}_{i}(t)\sigma^{\prime }({Inp}_{j}^{k}(t)).$$ (M17)

Again, this information is available at the synapse if we assume that the difference in activity between *M*_*j*_ and *C*_*j*_ is computed in the same cortical column as *k*_*j*_, which is common in models of predictive coding. When adding all the time steps, the total influence of *W*^*k*^_*ij*_ on *q*_*s*_*(t)* becomes:

$$\frac{\partial q_{s}(t)}{\partial W_{ij}^{k}}={Trace}_{ij}^{k}W_{sj}^{FB}.$$ (M18)

#### Tags and traces

RECOLLECT distinguishes between traces and tags (see also [16]). Whereas the traces represent the contribution of a synapse to the activity of the memory unit, the tags represent the influence of the synapse on the Q-value of the chosen action. The tag depends on the trace as well as on the amount of attentional feedback that arrives at the memory unit through the feedback connection from the chosen action (see Eqs 11 and 15).

The tags are used to implement the SARSA(λ) algorithm. If λ is larger than zero, the synapses that contributed to previous actions are also updated, while taking the temporal discount factor γ into account. This is an advantage of RECOLLECT and AuGMEnT [16] over e-prop [17], which uses a similar approach to approximating backpropagation-through-time. The resulting combination of tags and traces, can be shown to be equivalent to gradient descent through backpropagation-through-time on the temporal difference error in the absence of recurrent connections (see [16] for more detail), and to approximate backpropagation-through-time when recurrent weights are included.

### AuGMEnT architecture

This section explains the architecture of the AuGMEnT model (Rombouts et al., 2015) and how it differs from RECOLLECT.

AuGMEnT trains networks with three layers: an input layer, an association layer and a Q-value layer. The input layer consists of instantaneous units and transient units. The instantaneous units encode stimuli in the current timestep, and transient units signal changes in the stimuli. On-units become active if a stimulus appears and off-units if it disappears. The association layer also contains regular units and memory units, which exclusively receive information from instantaneous units and transient units, respectively. The activity of regular units depends on the input received at the current timestep, whereas memory units maintain information about stimuli presented during previous timesteps. Memory units of AuGMEnT lack a gating mechanism to block new sensory information or remove previous memory content. Consequently, memory content in AuGMEnT has to be erased at the end of a simulated trial because the learning rule cannot learn to forget the information in memory from the previous trial when a new trial starts. Both instantaneous and memory units project to Q-value units in the output layer of AuGMEnT, just as in RECOLLECT (see Eq 4). The learning rule in AuGMEnT is similar to that of RECOLLECT (see section ‘learning rule’ of the Results).
